# Pleurésie massive après chirurgie du cancer de sein et arrêt précoce du Tamoxifène: à propos d’‘une observation

**DOI:** 10.11604/pamj.2014.17.129.3837

**Published:** 2014-02-24

**Authors:** Léon Kabamba Ngombe, Ignace Bwana Kangulu, Chantal Mwenze Nday, Migrette Ngalula Tshanda, John Ngoy Lumbule, Pierre Mbayo Matanga, Olivier Ngoy Sampatwa, Michel Kabamba Nzaji

**Affiliations:** 1 Université de Kamina, Faculté de Médecine, Département de Médecine Interne, Kamina, République Démocratique du Congo; 2 Université de Kamina, Faculté de Médecine, Département de Gynécologie et Obstétrique, Kamina, République Démocratique du Congo; 3 Université de Lubumbashi, Lubumbashi, République Démocratique du Congo; 4 Université de Kamina, Faculté de Médecine, Département de Chirurgie, Kamina, République Démocratique du Congo; 5 Université de Kamina, Faculté de Médecine, Département de Santé Publique, Kamina, République Démocratique du Congo

**Keywords:** Pleurésie massive, cancer du sein, Tamoxyfène, massive pleural effusion, breast cancer, Tamoxyfen

## Abstract

Nous rapportons un cas de pleurésie massive droite probablement métastasique accompagnée d'un lymphœdème du membre supérieur droit ayant fait suite à une mastectomie et curage ganglionnaire indiqués pour un carcinome lobulaire du sein droit, associée à un arrêt précoce de la prise de Tamoxifène, vécu à Lubumbashi.

## Introduction

Le cancer du sein demeure un problème important de santé publique et une cause majeure de décès féminin dans le Monde [1]. Sur 100 femmes qui meurent d′un cancer, 25 ont un cancer du sein. Il est de loin le cancer le plus fréquent et le plus meurtrier de la femme. La prévalence est de plus de 4,4 millions de femmes. Les taux les plus élevés sont observés dans les pays industrialisés (Amérique du Nord, Australie, Europe de l'Ouest) et sont cinq fois plus élevés que dans les pays en voie de développement (Asie, Afrique) [2, 3].

La pleurésie constitue un motif fréquent de consultation en pneumologie. Ainsi, les pleurésies néoplasiques sont définies par la présence des cellules tumorales dans l'espace pleural. On estime que 20 pourcent de 150.000 patients qui décèdent de cancer annuellement en France sont porteurs d'une pleurésie néoplasique. Le cancer du sein et du poumon en France sont responsable à eux seuls d'environ 50 pourcents des pleurésies néoplasiques [4].

En République Démocratique du Congo en général et dans notre ville de Lubumbashi en particulier, nous n'avons pas de statistiques sur l’épidémiologie du cancer du sein ainsi que les pleurésies néoplasiques.

Nous rapportons dans ces papiers un cas de pleurésie massive survenue en post-opératoire lointaine, après arrêt précoce de la prise de Tomoxifène, d'une mastectomie avec curage ganglionnaire chez une femme indiquée pour carcinome lobulaire infiltrant du sein droit.

## Patiente et observation

Nous avons consulté une patiente, âgée de 44 ans, d'identité obstétricale P4G4D0A0, ménopausée à 40 ans et ayant comme plaintes: Dyspnée, douleur à l'hémothorax droit, tuméfaction du membre supérieur droit. Elle s'est automédiquée à l'ibuprofène et le non amendement des signes l'a poussé à consulter.

Dans les antécédents médico-chirurgicaux: En 2009, malade opérée pour un nodule du sein droit; En 2010, Récidive de la masse, suspicion à l’échographie d'un adénocarcinome du sein droit; De 2010 à 2012: Mammectomie totale du sein droit associée à une cure ganglionnaire dont l'anatomie pathologique a révélé un carcinome lobulaire infiltrant. Et traitement au Tamoxifène, 20 mg /j qu'elle a interrompu à 2 ans.

**Figure 1:**
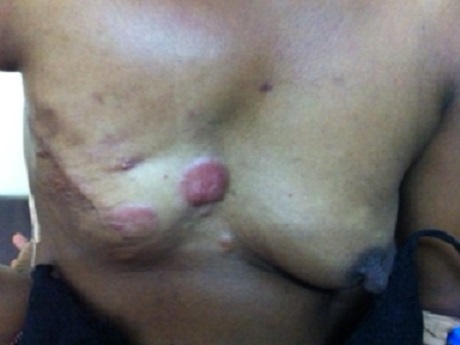
Tuméfaction d'environ 2 cm de diamètre dans la région du sein droit avec infiltration environnant

**Figure 2:**
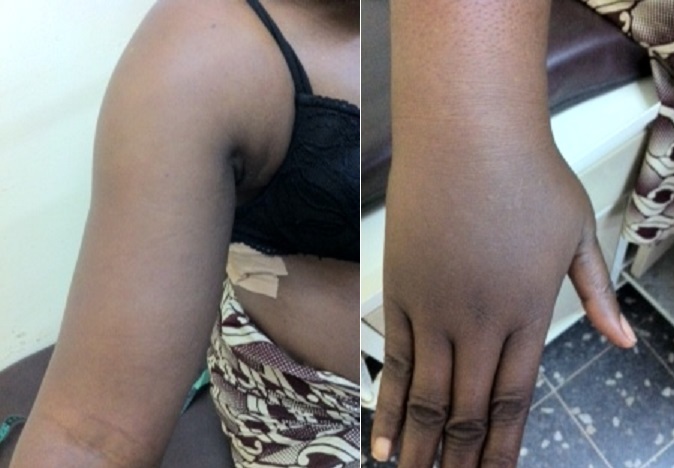
lymphœdème du membre supérieur droit

A l'examen physique, le thorax n’était pas ample et symétrique, on a noté la présence d'une tuméfaction d'environ 2 cm de diamètre dans la région du sein droit avec infiltration environnant; présence d'une matité et abolition des murmures vésiculaires à l'hémi thorax droit (Figure 1). Nous avons aussi noté une tuméfaction de tout le membre supérieur droit (lymphœdème) comparativement à celui opposé (Figure 2).

Au vu de tous ces éléments, nous avons posé le diagnostic d'une pleurésie probablement néoplasique secondaire à un cancer du sein associée à un lymphœdème suite.

**Figure 3:**
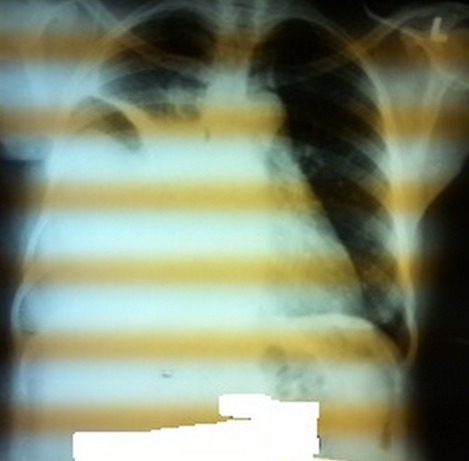
Images radiologiques de la pleurésie massive droite

La radiographique du thorax a confirmé la pleurésie (Figure 3) et deux ponctions évacuatrices ont été effectuées pour soulager la dyspnée. Le liquide de ponction était hématique. Les métastases osseuses, pelviennes et hépatiques n'ont pas été retrouvées.

## Discussion

La littérature montre que le cancer du sein est l'un des problèmes majeurs de santé publique dans les pays industrialisés. C'est la pathologie cancéreuse la plus fréquente chez la femme, une des premières causes de mortalité féminine dans le monde. L'Asie et l'Afrique ont des incidences basses [5]. Cependant dans notre milieu, les données ne sont pas disponibles et la prise en charge n'est pas toujours accessible.

La patiente reçue a présenté des dyspnées, la douleur à l'hémithorax droit et une tuméfaction du membre supérieur homolatéral. L'examen physique a mis en évidence un thorax asymétrique, présence d'une matité et abolition des murmures vésiculaires à droite, ce qui signe une pleurésie massive droite, confirmée par la radiographie thoracique. Cependant, la ponction pleurale a révélé l'aspect hématique du liquide d’épanchement. Une étude cytologique devrait être faite pour confirmer la notion de malignité.

Les métastases pulmonaires et pleurales ont été notées par plusieurs auteurs dans l′évolution d'un cancer du sein. En cas de cancer du sein, il est possible qu'il y ait un envahissement de la plèvre viscérale par voie hématogène ou par contiguïté, bloquant les voies de résorption du liquide pleural avec blocage des systèmes lymphatiques. Ceci pourrait expliquer la présence de la pleurésie et du lymphœdème du membre supérieur droit de notre observation. 65 pourcent des pleurésies malignes sont représentées par les métastases de cancer du poumon (40-45 pourcent) et du sein (20 pourcent) [4].

Il est également important de savoir que les métastases constituent la grande majorité des tumeurs pleurales, et par conséquent, elles représentent la première cause d’épanchement pleural après 50 ans [6]. La pleurésie, le lymphœdème et les infiltrations au niveau de la cicatrice opératoire, dans la région sous claviculaire droite apparus une année après l'arrêt de l'utilisation du Tamoxifene sont probablement une conséquence directe des métastases ou du cancer du sein lui-même.

Le lymphæ dème secondaire du membre supérieur après traitement du cancer du sein est une complication dont la fréquence dans la littérature est mal appréciée en raison des différentes définitions du lymphœdème [7, 8].

Sa fréquence après traitement du cancer du sein est estimée entre14 et 28%. De nombreuses études se sont intéressées aux facteurs de risque de développement de cette complication après traitement de ce cancer. Le nombre de ganglions enlevés lors du curage axillaire et la radiothérapie externe, en particulier sur le creux axillaire, sont les deux principaux facteurs de risque. Plusieurs autres facteurs de risque sont mineurs [9]. Dans le cas concerné, la technique chirurgicale pourrait être à la base.

Le Tamoxifène administré pendant 5 ans réduit de 40% le risque relatif de récidive et de 34% le risque relatif de décès. Le gain absolu à 15 ans est de 9,2% chez plus de 10000 malades randomisées: 25,6% de décès dans le groupe Tamoxifène contre 34,8% dans le groupe contrôle (méta-analyse sur données individuelles. Le bénéfice du Tamoxifène n'est influencé ni par l’âge, ni par l'envahissement ganglionnaire axillaire, ni par l'administration de la chimiothérapie [10]. La question d'une durée d'administration au-delà de 5 ans reste posée: chez 11500 malades randomisées dans l’étude ATLAS, le risque de récidive paraît moindre avec 10 ans de Tamoxifène qu'avec 5 ans (RR = 0,866 ± 0,048) [11]. Malheureusement, notre malade n'avait interrompu ce traitement qu’à deux ans, ce qui peut justifier la récidive tumorale et cette pleurésie hématique massive, signe fort probable de métastases pleurales.

Ce traitement est recommandé par la HAS (haute autorité de santé) dans le traitement du carcinome mammaire soit en traitement adjuvant, soit dans des formes évoluées avec progression locale ou métastase chez les femmes pré et ménopausées [12, 13], chose non respectée par la malade.

## Conclusion

Les métastases pleurales sont fréquentes dans les suites d′ un cancer de sein mais cependant, dans les pays sous équipés comme le nôtre, les moyens de diagnostic et de prise en charge efficaces posent problème. Même diagnostiqué à temps, les patientes n'ont pas souvent les moyens financiers pour soit entamer, soit achever le traitement recommandé vues le bas niveau socio-économique.
